# Henipaviruses and Fruit Bats, Papua New Guinea

**DOI:** 10.3201/eid1904.111912

**Published:** 2013-04

**Authors:** Hume Field, Carol E. de Jong, Kim Halpin, Craig S. Smith

**Affiliations:** Queensland Centre for Emerging Infectious Diseases, Brisbane, Queensland, Australia (H.E. Field, C.E. de Jong, C.S. Smith);; Life Technologies, Singapore (K. Halpin)

**Keywords:** viruses, henipavirus, pteropid bats, fruit bats, Papua New Guinea, emerging disease, Hendra, Nipah, bat, Australia

**To the Editor:** In 2010, detection of henipavirus (Hendra or Nipah virus) and rubulavirus (Tioman or Menangle virus) antibodies in fruit bats in Papua New Guinea (PNG) was reported (*1*). To explore changes in henipavirus dynamics in fruit bats, we compare and contrast this finding with serologic findings from 10 years earlier (*2*; H. Field et al., unpub. data).

In these earlier studies, blood samples were collected from 182 wild-caught fruit bats of mixed species, age, and sex from 3 locations in PNG: Madang (1996), New Britain (1997), and Lae (1999 ) (*2*; H. Field et al., unpub. data) (Figure). The 20 samples from Madang were collected as blood spots on filter paper and forwarded to the (then) Department of Primary Industries Animal Research Institute in Brisbane, Australia, where they were eluted and screened by ELISA for antibodies against Hendra virus (*3*). Serum from 59 samples from New Britain and 103 from Lae were forwarded to the Commonwealth Scientific and Industrial Research Organisation’s Australian Animal Health Laboratory in Geelong, Australia. Samples from New Britain were screened for antibodies against Hendra virus by virus neutralization test (VNT) (*3*). Positive samples were subsequently screened by VNT for antibodies against Nipah virus. Samples from Lae were screened by VNT for Hendra, Nipah, and Menangle viruses (*3*). A reciprocal VNT titer of >5 was considered indicative of antibodies.

**Figure:**
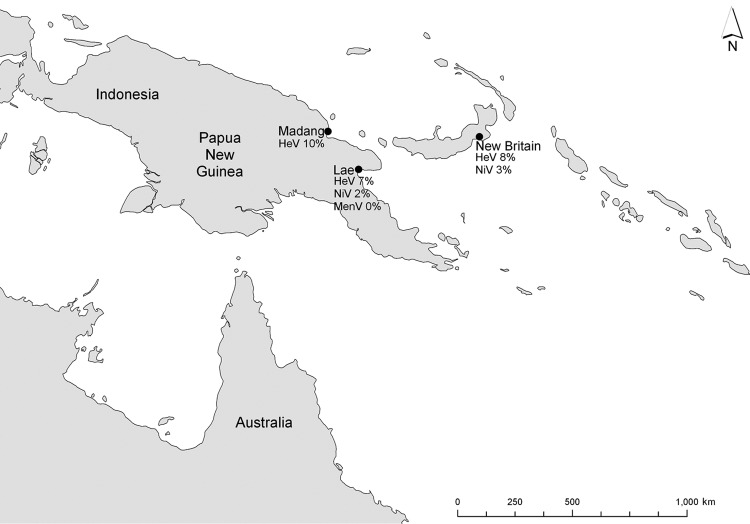
Sampling locations and henipavirus antibody prevalence, Papua New Guinea 1996–1999, among 182 wild-caught fruit bats from Madang (1996), New Britain (1997), and Lae (1999), Papua New Guinea. HeV, Hendra virus; NiV, Nipah virus; MenV, Menangle virus.

Of the 20 samples from Madang, 2 (10%) reacted in the Hendra virus ELISA. Of the 147 samples from New Britain and Lae that yielded definitive VNT results, 11 (7.5%) yielded neutralizing antibodies to Hendra virus and 5 (3.4%) to Nipah virus. All samples with antibodies against Nipah virus also had antibodies against Hendra virus; titers against Hendra virus were greater (4 samples) or equivalent (1 sample) to those against Nipah virus. Reciprocal titers against Hendra virus were 5–160 (median 10) and against Nipah virus, 5–80 (median 10). None of the 103 samples from Lae had antibodies against Menangle virus (Figure).

The common and contrasting findings between the study of Breed et al. (*1*) and the earlier studies are as follows. First, the earlier studies identified antibodies against Hendra virus in fruit bats from multiple locations and species, as did the study by Breed et al. (*1*). However, in marked contrast, the earlier studies found a crude prevalence of antibodies against Hendra virus of 7.8% (13/167) compared with 50% found by Breed et al. (*1*). Although this difference could reflect confounding by species, location, or time, when we controlled for the first 2 by comparing only 1 bat species (*Pteropus conspicillatus*) from proximate locations (Lae and Madang), the significant differences in antibody prevalence remained: 0 (95% CI 0–23%) in 1999 and 65% (95% CI 50%–78%) in 2009.

Second, in the earlier studies, all bats (except 1) that had a neutralizing antibody titer to Nipah virus had a higher neutralizing titer to Hendra virus (1 bat had equivalent titers), suggesting that the circulating henipavirus was more similar to Hendra virus than to Nipah virus. These findings are supported at a regional level by those reported for nearby Indonesian islands by Sendow et al. (*4*). However, the more recent findings of Breed et al. (*1*) suggest the opposite. Of note, in the earlier PNG studies, titers against Hendra and Nipah viruses were modest (median 10) and pose the possibility of cross-neutralization by a related, but undescribed, additional henipavirus.

Third, the earlier studies found no antibodies against Menangle virus in the 103 samples; in contrast, Breed et al. found 56% (*1*). Thus, earlier studies found no samples with antibodies against Menangle virus and henipavirus, in contrast to 36% of samples reported by Breed et al. (*1*).

Although any of these differences might have multiple interpretations, the collective scope and magnitude of the differences is more consistent with a major 10-year change in infection dynamics in these bat populations. Sendow et al. (*4*), when reporting henipavirus infections in fruit bats in Indonesia, canvassed the geographic extent of Hendra virus and Nipah virus, concluding that a transition probably occurred between Hendra virus in Australia and Nipah virus in Malaysia and beyond. They also concluded that further research was needed to understand the nature and stability of the interface between Hendra virus and Nipah virus and to investigate the possible presence of unidentified cross-neutralizing henipaviruses.

Changed henipavirus dynamics in PNG fruit bat populations could reflect altered population dynamics (and consequent infection dynamics) associated with negative ecologic effects (e.g., habitat loss, encroachment) (*5*). More broadly, such changes might portend a regional shift in the geographic interface between Hendra and Nipah virus endemicity.

More robust interpretation of the serologic findings of both studies is constrained by the lack of henipavirus sequence data from PNG and neighboring countries. We concur with Breed et al. (*1*) that sequencing and phylogenetic analyses are imperative if the ecology of henipaviruses in fruit bat populations and the implications for human and livestock health in the region are to be fully understood.

## References

[1] Breed AC, Meng Y, Barr JA, Crameri G, Thalmann CM, Wang LF. Prevalence of henipavirus and rubulavirus antibodies in pteropid bats, Papua New Guinea. Emerg Infect Dis. 2010;16:1997–9. 10.3201/eid1612.100879 21122242 PMC3294587

[2] Mackenzie JS. Emerging viral diseases: an Australian perspective. Emerg Infect Dis. 1999;5:1–8. 10.3201/eid0501.990101 10081666 PMC2627694

[3] Daniels P, Ksiazek T, Eaton B. Laboratory diagnosis of Nipah and Hendra virus infections. Microbes Infect. 2001;3:289–95. 10.1016/S1286-4579(01)01382-X 11334746

[4] Sendow I, Field HE, Curran J. Darminto, Morrissy C, Meehan G, et al. Henipavirus in Pteropus vampyrus bats, Indonesia. Emerg Infect Dis. 2006;12:711–2. 16715584 10.3201/eid1204.051181 PMC3294701

[5] Mickleburgh S, Hutson A, Racey P. Old World fruit bats: an action plan for their conservation. Gland (Switzerland): International Union for Conservation of Nature; 1992.

